# Skeletal Characterization of Smurf2-Deficient Mice and *In Vitro* Analysis of Smurf2-Deficient Chondrocytes

**DOI:** 10.1371/journal.pone.0148088

**Published:** 2016-01-27

**Authors:** Henry Huang, Eric S. Veien, Hong Zhang, David C. Ayers, Jie Song

**Affiliations:** 1 Department of Orthopedics and Physical Rehabilitation, University of Massachusetts Medical School, Worcester, MA, United States of America; 2 Department of Cell and Developmental Biology, University of Massachusetts Medical School, Worcester, MA, United States of America; University of Alabama at Birmingham, UNITED STATES

## Abstract

Overexpression of Smad ubiquitin regulatory factor 2 (Smurf2) in chondrocytes was reported to cause spontaneous osteoarthritis (OA) in mice. However, it is unclear whether Smurf2 is involved in bone and cartilage homeostasis and if it is required for OA pathogenesis. Here we characterized age-related changes in the bone and articular cartilage of Smurf2-deficient (MT) mice by microCT and histology, and examined whether reduced Smurf2 expression affected the severity of OA upon surgical destabilization of the medial meniscus (DMM). Using immature articular chondrocytes (iMAC) from MT and wild-type (WT) mice, we also examined how Smurf2 deficiency affects chondrogenic and catabolic gene expressions and Smurf2 and Smurf1 proteins upon TGF-β3 or IL-1β treatment in culture. We found no differences in cortical, subchondral and trabecular bone between WT and MT in young (4 months) and old mice (16–24 months). The articular cartilage and age-related alterations between WT and MT were also similar. However, 2 months following DMM, young MT showed milder OA compared to WT (~70% vs ~30% normal or exhibiting only mild OA cartilage phenotype). The majority of the older WT and MT mice developed moderate/severe OA 2 months after DMM, but a higher subset of aged MT cartilage (27% vs. 9% WT) remained largely normal. Chondrogenic gene expression (Sox9, Col2, Acan) trended higher in MT iMACs than WT with/without TGF-β3 treatment. IL-1β treatment suppressed chondrgenic gene expression, but Sox9 expression in MT remained significantly higher than WT. Smurf2 protein in WT iMACs increased upon TGF-β3 treatment and decreased upon IL-1β treatment in a dose-dependent manner. Smurf1 protein elevated more in MT than WT upon TGF-β3 treatment, suggesting a potential, but very mild compensatory effect. Overall, our data support a role of Smurf2 in regulating OA development but suggest that inhibiting Smurf2 alone may not be sufficient to prevent or consistently mitigate post-traumatic OA across a broad age range.

## Introduction

Transforming growth factor-β (TGF-β) signaling consists of multiple secreted ligands such as bone morphogenic proteins (BMPs), TGF-βs, activins, inhibins, and growth and differentiation factors (GDFs) that regulate many cellular processes including proliferation, differentiation and apoptosis. Its involvement in limb formation is tightly regulated in order to ensure proper development and maintenance of bone and cartilage tissues [1]. For instance, TGF-β signaling is essential for joint homeostasis by promoting cartilage matrix synthesis [2] and preventing chondrocytes from undergoing terminal differentiation [3]. Aberrations in the TGF-β/BMP signaling have thus been associated with many skeletal disorders such as osteoporosis, heterotopic ossifications, and osteoarthritis (OA) [4, 5]. For instance, TGF-β1 and TGF-β3 are shown to be diminished in human and mouse OA cartilage, respectively [6, 7], and transgenic mice that lose TGF-β signaling in cartilage recapitulate an OA-like phenotype [8–10].

One way in which TGF-β/BMP signaling is regulated is through the ubiquitin system. Ubiquitination is a post-translational modification that requires the step-wise effort of E1 activating enzymes, E2 conjugating enzymes and E3 ubiquitin ligases. Ubiquitinated proteins are typically known to be targets for proteasomal degradation [11], however, non-degradative roles have also been reported which can alter a protein’s function [12] or its localization [13]. Smad ubiquitin regulatory factor 1 and 2 (Smurf1 and Smurf2) are E3 ubiquitin ligases that share high homology and have been shown in various cell types to regulate TGF-β/BMP signaling [14]. They inhibit TGF-β signaling by promoting the degradation of R-Smads (Smads 1, 2 and 3) and TGF-β receptors [14, 15].

Based on mouse models, Smurf1 has been implicated in various signaling pathways associated with bone development and function [16]. Smurf1-deficient mice are phenotypically normal at birth, but exhibit an age-dependent increase in cortical bone mass due to sensitization of Smurf1-deficient osteoblasts to BMP [17]. Smurf2 has been shown to be upregulated in cartilage explants from OA patients [18]. Using a transgenic mouse model, Wu *et al*. demonstrated that overexpression of Smurf2 in articular chondrocytes results in chondrocyte hypertrophy and accelerated cartilage degradation [18]. The potential of Smurf2 to trigger spontaneous cartilage degradation raises the questions as to what roles Smurf2 has during normal cartilage development and maintenance and whether aberrant expression of Smurf2 is required for OA development. Smurf2-deficient mice [19, 20] are born at the expected Mendelian ratio with no obvious phenotypic abnormality, but an increased incidence of tumor formations at 15–20 months compared to wild-type (WT). To our knowledge, there has been no in-depth characterization of age-dependent changes in bone and cartilage in mice with reduced Smurf2 expression.

**Fig 1:**
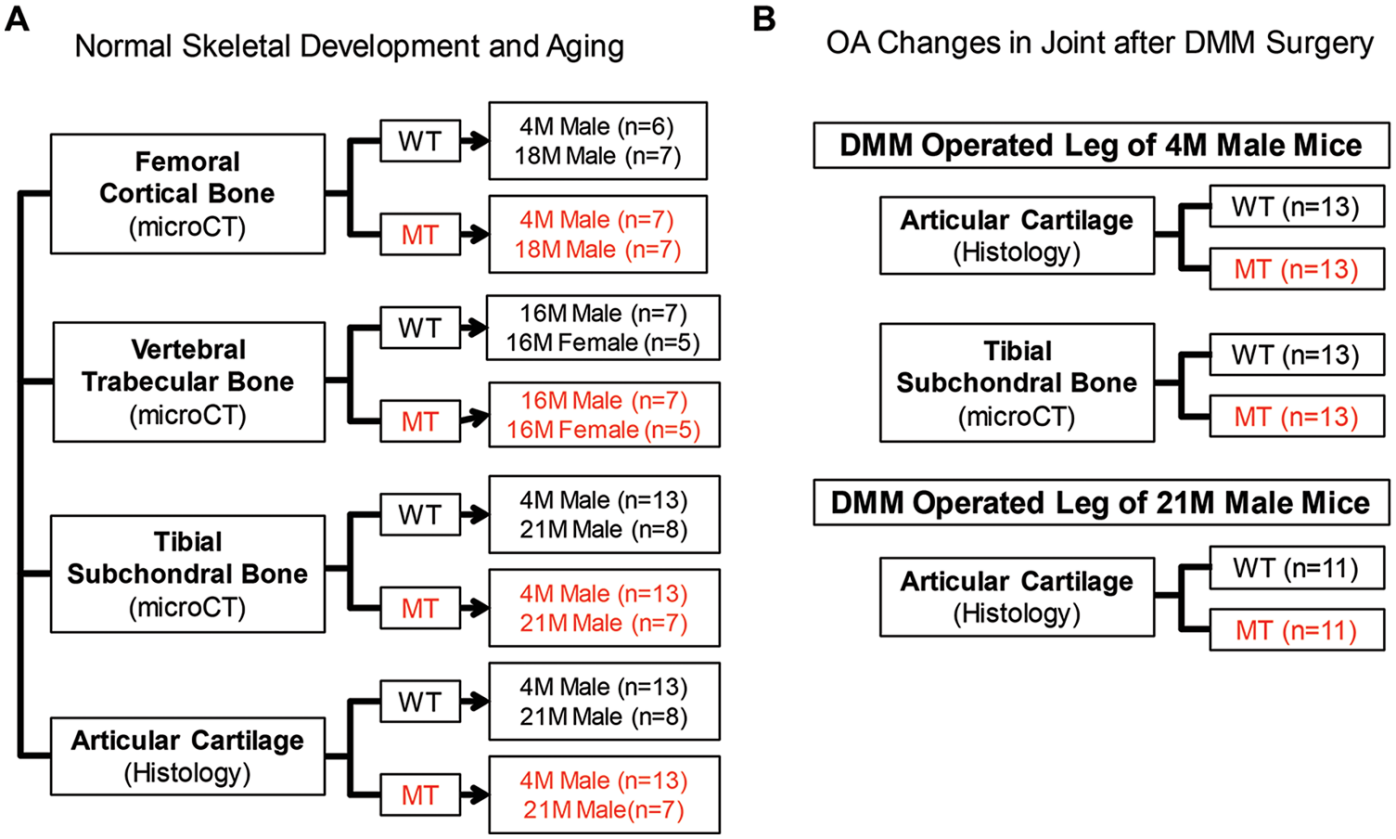
Summary of experimental design for skeletal characterizations.

Here, we used a Smurf2-deficient mouse model generated by gene trapping [19] to investigate whether reduced Smurf2 expression affects normal skeletal development and aging and whether it affects pathological conditions such as post-traumatic OA. Specifically, we characterized femoral cortical bone, vertebral trabecular bone, knee subchondral bone and knee articular cartilage of skeletally mature young (4 months old) and old (21 ± 1.3 months) Smurf2-deficient (MT) mice and their WT counterparts by quantitative microcomputed tomography (microCT) analyses and semi-quantitative histological scoring of articular cartilage (Fig 1A). In addition, we assessed the severity of OA symptoms of WT vs. MT mice in both age groups in response to surgical destabilization of the medial meniscus (DMM) (Fig 1B). Lastly, we utilized primary immature chondrocytes isolated from WT and MT mice to assess how Smurf2 deficiency affects chondrogenic and catabolic gene expression as well as Smurf2 and Smurf1 protein expression in 2D culture after treatment with TGF-β3 or pro-inflammatory cytokine IL-1β.

## Materials and Methods

### Breeding of Smurf2-deficient and WT Mice

A previously reported Smurf2-deficient mouse model generated by gene trapping (on a C57BL/6 background) [19] that was developed in the Zhang lab at University of Massachusetts Medical School (Worcester, MA) was used for the study. WT and MT mice were generated by crossing mice heterozygous for the trapped *Smurf2* allele. For isolation of primary chondrocytes, homozygous mice were bred to produce sufficient neonates of the same genotype. Adult mice and neonates were euthanized by carbon dioxide asphyxiation followed by either cervical dislocation (adults) or decapitation (neonates). All mice were housed in a fully accredited Animal Care facility and the animal handling and surgical procedure were approved by the University of Massachusetts Medical School Institutional Animal Care and Use Committee (IACUC).

### Bone Marrow Stromal Cells (BMSC) Isolation

Hind legs were harvested from skeletally mature 4 months old WT and MT mice. Excess muscles were trimmed off and one end of the tibiae and femur was cut. Adopting a literature method [21], bone marrow (BM) plugs were isolated by inserting a 25-gauge syringe needle into the uncut end of the long bones and flushing with serum-free α-MEM. The BM plugs were mechanically disrupted using a pipettor and filtered through a 70-μm mesh to remove excess debris. Red blood cells were lysed by mixing with an equal part of sterile water for <10 sec followed by a 1:5 dilution in 1×PBS. Cells were pelleted at *400g* for 10 min and resuspended in complete culture media (α-MEM with 20% hyclone FBS, 1% L-glutamine, and 1% pen/strep). The total cell suspension was plated on two p100 plates and media were changed twice a week.

### Immature Murine Articular Chondrocyte (iMAC) Isolation

Following a literature protocol [22], iMACs were isolated from 5 to 7 day-old neonates. Cartilage from the knee and ankle joints were harvested, pooled together and subjected to a 60-min digestion in type II collagenase (Worthington) solution (3mg/mL in high glucose DMEM with 1% pen/strep) at 37°C under agitation. The cartilage pieces were then transferred to a diluted collagenase solution (0.5mg/mL) and incubated for 5–6 hours at 37°C under moderate agitation. The solution with residual fragments were mixed to yield a single cell suspension and filtered through a 70-μm nylon mesh. The chondrocytes were pelleted at *400g* for 10 min, washed in PBS and then resuspended in DMEM/F12 with 10% FBS. The cells were plated at a density of 2.5 x 10^4^ cells/cm^2^ and cultured for 4–6 days before subsequent *in vitro* treatments and analyses. The chondrogenic nature of these cells was confirmed by positive Alcian Blue staining for cartilage ECM and the expression of chondrogenic gene which decreased with multiple passaging.

### Whole Tissue Dissection

The hind legs from 4 months old WT and MT were harvested and each leg was dissected into 1) muscles surrounding the tibia and femur, 2) knee joint including the patella, ligaments and the tibial and femoral articular ends, and 3) mid-shaft tibia and femur with intact bone marrow. The spleen was harvested as a control where Smurf2 is known to be highly expressed in the WT. All specimens were immediately frozen in liquid nitrogen, powderized using a mortar and pestle, and stored at -80°C until time for protein or RNA isolation.

### Western blot for BMSCs, iMACs, and musculoskeletal tissues

Total cell lysates from Passage 0 BMSCs and iMACs were extracted using RIPA buffer (50mM Tris pH 7.5, 150mM NaCl, 1% Triton X, 1mM EDTA, 0.1% SDS, 0.5% Na-deoxycholate) supplemented with a protease inhibitor cocktail (Roche). For protein isolation from whole bone, joint and skeletal muscle, powderized specimens were resuspended in RIPA. Protein lysates (25–60μg) were separated by SDS-PAGE mini-protean gels (Bio-Rad) and transferred to nitrocellulose membranes. Membranes were probed using primary antibodies against Smurf2 (Abcam, 1:1000), Smurf1 (Abcam, 1:2000), Col2 (Millipore, 1:2000), Sox9 (Millipore, 1:1000), GAPDH (Sigma, 1:5000), tubulin (Sigma, 1:5000) and goat secondary antibodies against rabbit (1:5000) and mouse (1:10,000) IgG and then visualized using a chemiluminescent substrate (Pierce).

### RT-PCR for Smurf2 mRNA levels in whole bone and cartilage tissues

Powderized tissues were resuspended in TRIzol reagent (Life Technologies) and vortexed at room temperature for 15–20 min. Insoluble components were removed by centrifugation (12,000*g*, 10 min). RNA was purified using Direct-zol RNA MiniPrep (Zymo Research) according to manufacturer’s instructions and RNA concentration was determined using a Nanodrop 2000 spectrophotometer (Thermo Scientific). Total RNA (1 μg) was reverse-transcribed using SuperScript III First-Strand Synthesis Kit (Life Technologies) according to manufacturer’s instructions. PCR reactions were conducted on a GeneAmp PCR system 2700 (Applied Biosystems) under the following conditions: 94°C for 3 min, followed by 35 cycles at 94°C for 20 sec, 58°C for 30 sec, 72°C for 1 min, and a final extension at 72°C for 10 min. PCR products were subjected to electrophoresis on a 1% agarose gel and visualized by ethidium bromide staining. RT-PCR primer sequences: Smurf2 (Fwd: 5’-AACCGTGCTCGTCTCTCTTC-3’, Rev: 5’-ATGAAGTCATTCCCCAGCAC-3’); GAPDH (Fwd: 5’-AGGTCGGTGTGAACGGATTTG-3’, Rev: 5’-TGTAGACCATGTAGTTGAGGTCA-3’.)

### Preparation of skeletal tissues for microCT and histology

For analysis of knee joints by microCT and histology, hind legs were harvested from skeletally mature young (4 months old) and old (21 ± 1.3 month old) male mice, tied to a toothpick in a fully extended position, and fixed in 10% neutral buffered formalin for 2–3 days at 4°C before being scanned and subsequently decalcified for histology. For analysis of femoral cortical bone and vertebral trabecular bone, hind legs from 4 and 18 month old mice and lumbar vertebrae from 16 month old male and female mice were harvested and fixed in 10% neutral buffered formalin for 2–3 days at 4°C before being scanned by microCT and subsequently decalcified for histology.

### Quantitative MicroCT analyses

All skeletal tissues were scanned on a Scanco microCT 40 scanner (Scanco Medical, Brüttisellen, Switzerland) at a 10-μm voxel resolution. Cortical bone analysis was performed by evaluating 50 slices (volume of interest/VOI) from the midshaft (10 micron spacing, flanking the midpoint between growth plates of the femur) for bone volume, cortical thickness, and bone mineral density. Vertebral bone analysis was performed on either the L4 or L5 vertebrae and the region of interest (ROI) was defined by the entire trabeculae within the body of the vertebra excluding the cortical bone (S1A Fig). The bone volume/tissue volume (BV/TV), trabecular thickness, trabecular spaces, and bone mineral density were determined using Scanco’s trabecular bone evaluation that is based on distance transformations (Direct-No model method). For subchondral bone analysis, the medial and lateral condyles of the proximal tibia epiphysis were analyzed separately. The ROI was defined by the bone between the calcified cartilage and the growth plate (S1B Fig). The bone volume/tissue volume (BV/TV), trabecular thickness, trabecular spaces, and bone mineral density were calculated using the same trabecular bone evaluation method as the vertebrae. For subchondral bone analysis after DMM surgery, only the medial condyle of the tibia was evaluated and osteophytes were excluded from the ROI.

### Knee Histology and Semi-Quantitative Scoring of Articular Cartilage

Formalin-fixed hind legs were decalcified for 14 days in 18% EDTA and frontally embedded in paraffin blocks. Blocks were cut using a Reichert-Jung 2030 microtome to produce 5μm thick sections. Sections were deparaffinized then stained with Weigert’s iron hematoxylin/fast green/safranin-O (Sigma). To systematically compare knee articular cartilages, a minimum of five equally spaced sections spanning the knee joint were analyzed using a modified literature semi-quantitative scoring criteria [23]. Articular cartilage with loss of staining or superficial structural damage was referred to as mild OA (scores of 0.5 and 1), while fibrillations and erosions with increasing depth and width below the superficial layer and extending past tidemark were referred to as moderate (scores of 2 and 3) or severe OA (score of 4). Sections with majority of the subchondral bone exposed or only a small portion of articular cartilage intact were categorized as very severe OA (scores of 5 and 6). The medial femoral and tibial articular cartilages of each knee section were blindly scored by 7 trained examiners. The scores from each examiner were averaged and the average scores for the femoral and tibial cartilage were plotted separately on the same graph for semi-quantitative comparisons.

### Surgical Induction of OA by Destabilization of Medial Meniscus (DMM)

DMM, a well-established surgical model for inducing OA [24], was performed on 2 months old and 19 ± 1.3 month old male mice. Briefly, the mice were anesthetized using 2% isoflurane in oxygen, and the surgical site was sterilized before a medial parapatellar incision was made to expose the joint cavity. The meniscotibial ligament was identified and transected under a stereomicroscope. Joint capsule and overlying skin were sutured in layers using 7–0 PGA sutures. Buprenorphine (0.05mg/kg, 3 times a day) and cefazolin (20mg/kg, twice a day) were injected subcutaneously immediately post-operation (post-op) and for 2 more days thereafter. Mice were allowed to move freely in cages immediately after the operation. At 2 months post-DMM, the mice were euthanized and their hind legs were harvested and prepared for microCT and histology analyses as described above.

### Quantitative RT-PCR (qPCR) of Primary Chondrocytes

iMACs were isolated from WT and MT mice as described above. Cells were seeded at 2.5 x 10^4^ cells/cm^2^ and cultured in expansion media (5% FBS in DMEM/F12) for 4 days on tissue culture polystyrene (TCPS). Cells were then treated with 10 ng/mL of TGF-β3 (R&D Systems) or 5 ng/mL of IL-1β (R&D Systems) for 24 hours. RNA was isolated, purified and reverse-transcribed as described above. qPCR samples were prepared using Power SYBR Green Master Mix (Applied Biosystems) and analyzed using 7500 Real-Time PCR System (Applied Biosystems). Gene expressions of Sox9, Col2, Acan, Col1, MMP-3, MMP-9, MMP-13, and ADAMTS5 were normalized to β-actin levels. Data were plotted as fold change relative to those of untreated WT. Primer sequences are summarized in Table 1.

**Table 1:** Sequences of primers used for quantitative RT-PCR.

Gene	NCBI Ref Seq	Forward Primer Sequence 5’-3’	Reverse Primer Sequence 5’-3’	Primer Size (bp)
β-Actin	NM_007393.3	CGAGCGGTTCCGATGC	TGGATGCCACAGGATTCCAT	69
SOX9	NM_011448.4	AGGAAGCTGGCAGACCAGTA	CGTTCTTCACCGACTTCCTC	193
COL2	NM_031163.3	GATCACCTCTGGGTCCTTGTT	TCCTCTGCGATGACATTATCT	222
ACN	NM_007424.2	AGTGGATCGGTCTGAATGACAGG	AGAAGTTGTCAGGCTGGTTTGGA	105
COL1	NM_007742.3	AACGAGATCGAGCTCAGAGG	CACGAAGCAGGCAGGGCCAA	213
MMP-3	NM_010809	AGTCTACAAGTCCTCCACAG	TTGGTGATGTCTCAGGTTCC	152
MMP-9	NM_013599.3	TAGCTACCTCGAGGGCTTCC	GTGGGACACATAGTGGGAGG`	147
MMP-13	NM_008607.2	AGACCTTGTGTTTGCAGAGCACTAC	CTTCAGGATTCCCGCAAGAGT	70
ADAMTS5	NM_011782.2	CGAAGAGCACTACGATGCAG	TGGAGGCCATCATCTTCAAT	144

### Statistical Analysis

Statistical analyses were performed using Prism (Graphpad Software, Version 6.0). MicroCT data were presented as the mean ± standard deviation and the statistical analyses were performed using 2-way ANOVA followed by Tukey’s post hoc test. Knee histological scores were plotted as a dot plot with the median and interquartile range, and the statistical analyses were performed with either Kruskal-Wallis with Dunn’s multiple comparison test (for age and genotype-dependent changes) or the Mann-Whitney rank-sum test (response to DMM surgery). Distribution of the histological scores from post-DMM WT and MT knees were compared using Kolmogorov-Smirnov test. qPCR results are presented as a mean ± standard deviation of three separate chondrocyte isolation experiments for each genotype and the statistical analysis was performed using unpaired *t*-test for each treated and untreated condition. Quantifications of WB bands were normalized to WT expansion media control and averaged among three replicates. A value of *p* < 0.05 was considered statistically significant.

## Results

### Characterization of Smurf2 Expression in skeletal tissues

**Fig 2:**
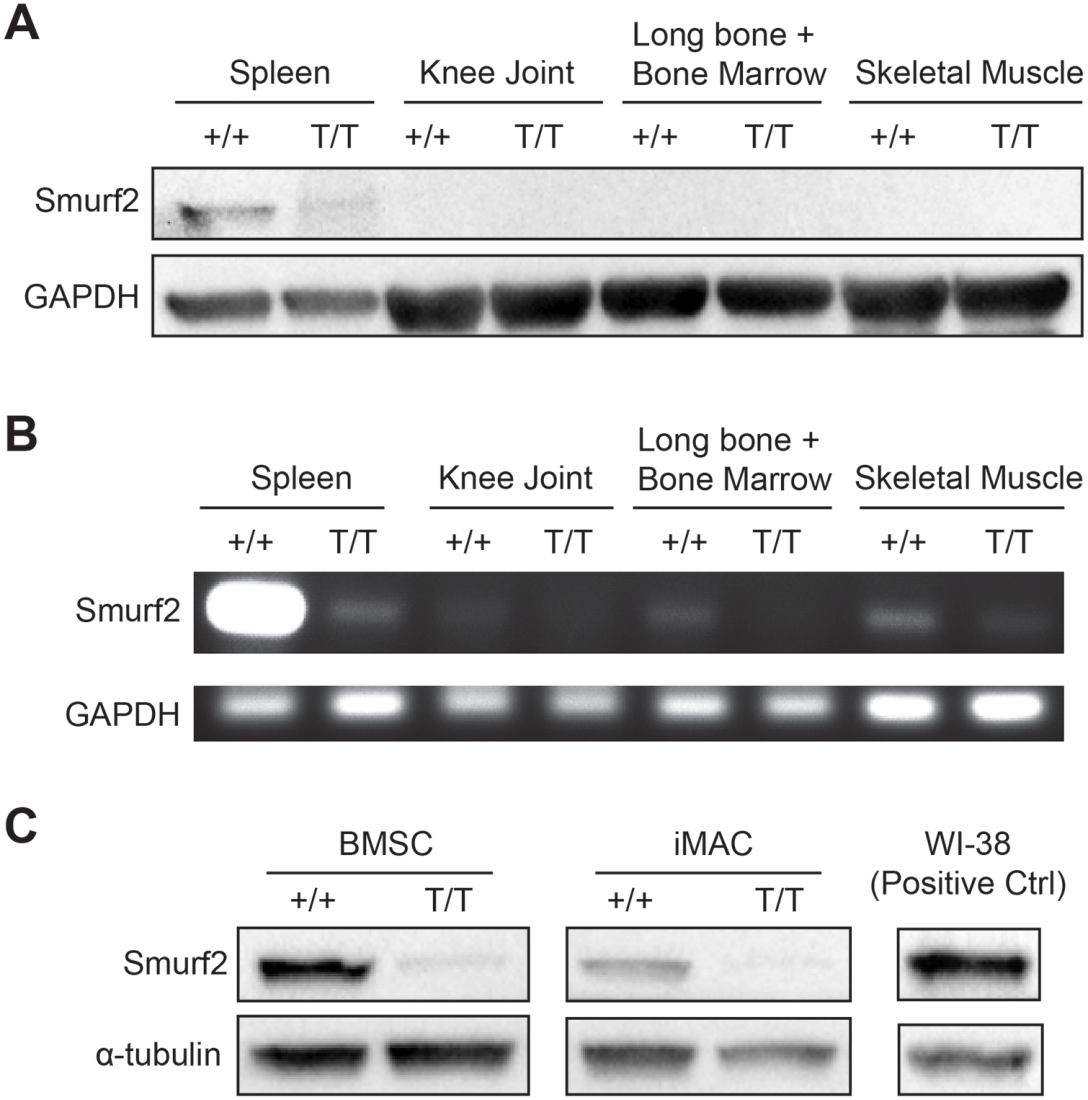
Smurf2 protein and gene expressions in WT (+/+) and Smurf2-deficient MT (T/T) skeletal tissues and primary cells. (A) Protein expression of Smurf2 in various skeletal tissues from healthy 4 month old male WT and MT mice. Spleen, where Smurf2 is highly expressed, is included as a positive control. (B) Smurf2 mRNA expression in various skeletal tissues compared to spleen in 4 month old male WT and MT mice. (C) Protein expression of Smurf2 in bone marrow stromal cells (BMSC, passage 0) isolated from 4 month old WT and Smurf2-deficient MT mice and immature articular chondrocytes (iMAC, passage 0) isolated from WT and MT neonates.

To study the role of Smurf2 in skeletal tissues, we began by examining the levels of Smurf2 expression in theses tissues. The level of Smurf2 protein in knee joints, cortical bones/bone marrow, and skeletal muscles of C57BL/6 WT mice was undetectable compared to levels in the spleen by western blot (Fig 2A). Using RT-PCR, we confirmed that the total mRNA levels of Smurf2 extracted from these skeletal tissues were much lower than in the spleen, but all MT tissue examined showed reduced Smurf2 mRNA expression compared to WT as expected (Fig 2B). We also examined Smurf2 expression in bone marrow stromal cells (BMSC) and immature murine articular chondrocytes (iMAC) isolated from MT and WT mice, and found that Smurf2 protein levels are significantly reduced in both MT primary cells (Fig 2C).

### Smurf2-deficient mice exhibit normal age-dependent cortical and trabecular bone phenotypes

**Fig 3:**
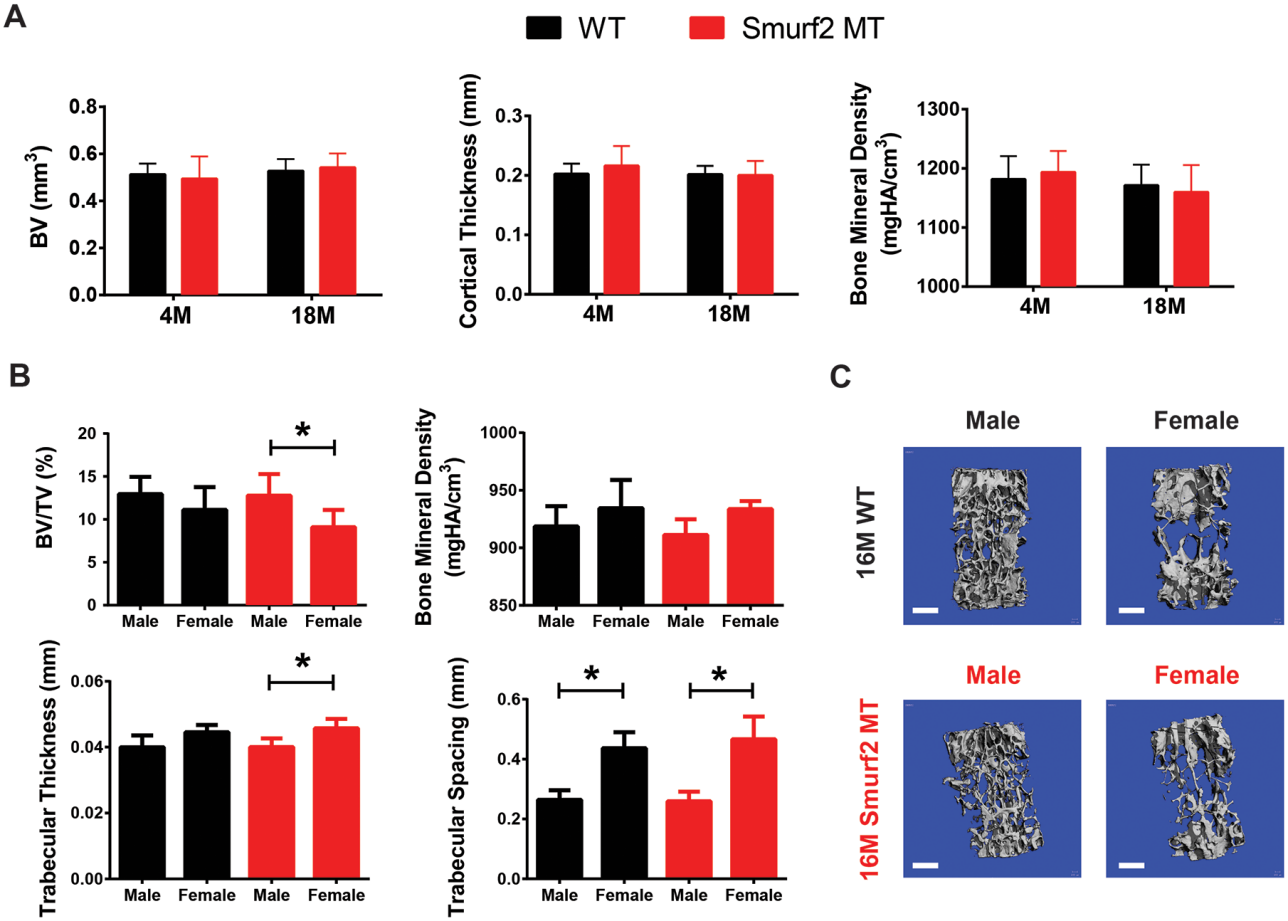
Age- and gender-specific cortical bone and trabecular bone analyses of WT and Smurf2-deficient MT mice. (A) Mid-shaft cortical bone analysis of male WT and MT femurs from 4 month (WT: n = 6; MT: n = 7) and 18 month old (WT and MT: n = 7) mice. (B) Gender-specific trabecular bone analysis of lumbar vertebrae from 16 month old WT and MT mice (Male: n = 7; Female: n = 5). (C) Representative 3D reconstruction of contoured vertebral trabecular bone. Scale bar = 500 μm.

Using microCT, we examined changes in mid-shaft femoral cortical bone between WT and MT mice at 4 months and 18 months of age. Although a slight drop in bone mineral density (BMD) was observed with advanced age, these changes were not statistically significant. No significant difference between WT and MT mice at a given age was detected (Fig 3A).

Due to its high surface area to bone matrix volume ratio, trabecular bone undergoes more rapid remodeling and is associated with more prominent bone loss with age than in cortical bone [25]. We therefore assessed the vertebral trabecular bone in 16 month old mice using microCT to reveal potential remodeling abnormality between genotypes. To account for the effect of estrogen deficiency on bone remodeling, these analyses were performed in a gender-specific manner (Fig 3B and 3C). Regardless of genotype, the vertebral trabecular bone of old female mice exhibited lower BV/TV and higher trabecular thickness and trabecular spaces than their male counterparts. These gender-specific differences were not statistically different between WT and MT mice.

### Smurf2-deficient mice exhibit normal knee joint phenotype

**Fig 4:**
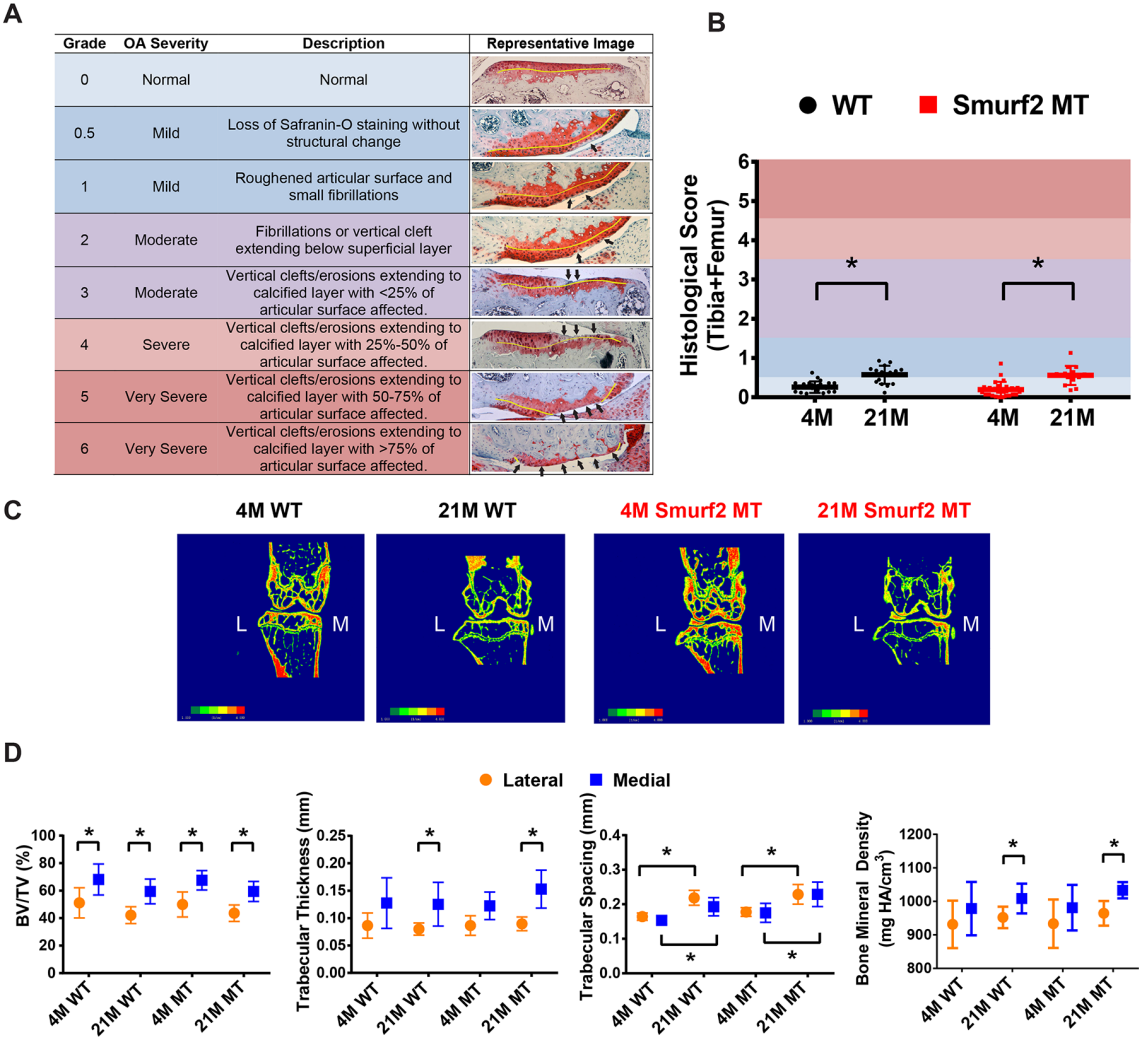
Age-specific knee joint phenotypes of male WT (+/+) and Smurf2-deficient MT (T/T) mice. (A) Normal and osteoarthritic joint articular cartilage histology scoring criteria modified over literature method[27] along with representative safranin O-stained cartilage sections. Yellow line indicates tidemark; Black arrows denote loss of staining, fibrillations, or erosions. (B) Combined histology scores of femoral and tibial articular cartilage of 4 month old WT (n = 13), 4 month old MT (n = 13), 21 month WT (n = 11), and 21 month old MT (n = 11). (C) Representative microCT images of bone mineral density color mappings of mid-frontal knee sections from young and old WT and Smurf2 MT mouse knees. Red indicates higher BMD while green indicates lower BMD. D) Quantitative comparisons of the lateral and medial subchondral bone analyses between 4 month (WT: n = 9; MT: n = 11) and 21 month (WT: n = 10; MT: n = 8) WT and MT mice. *p<0.05.

Qualitative comparison of histological sections of WT and MT knee joints did not reveal any gross developmental or age-related abnormalities within the growth plates, menisci, or synovium (S2 Fig). In order to characterize the structural changes in knee articular cartilage that would reflect spontaneous OA development with age, we adopted from literature a semi-quantitative knee histological scoring system [23] and categorized each score to an overall OA severity (Fig 4A). Since lateral tibial articular cartilage tends to be thinner with less distinct zonal architecture [26], we focused our analyses on the medial side. Using this scoring system, we detected age-dependent changes in the articular cartilage from normal to mild OA in the medial tibial and femoral condyles in both the WT (*p* = 0.0011) and MT (*p* < 0.0001) mice, but no statistically significant difference was detected between them at a given age (Fig 4B). The main differences observed between the two age groups ranged from general loss of proteoglycan staining to mild superficial fibrillations as reflected by the median scores of 0.64 (0.35–0.72) and 0.57 (0.41–0.67) in the 21 month old WT and MT groups, and 0.26 (0.14–0.36) and 0.14 (0.06–0.26) in the young 4 month old WT and MT groups, respectively.

We then investigated whether age-dependent changes in the tibial subchondral bone were different between WT and MT. Since previous work reported differences in the medial and lateral compartments of the subchondral bone [27], we analyzed microCT data in these two compartments separately. Compared to the lateral side, the medial subchondral bone compartment had a higher BV/TV, trabecular thickness and BMD across both age groups and genotypes (Fig 4C and 4D). The age-dependent loss of trabecular bone within the medial compartment was also apparent as supported by the increase in trabecular spacing, from 0.154±0.011 (WT) and 0.175±0.027 (MT) in the young group to 0.193±0.026 (WT) and 0.229±0.036 (MT) in the old group, respectively. However, no statistically significant differences were detected between WT and MT mice at a given age.

### Young Smurf2-deficient mice develop milder OA in knee articular cartilage compared to WT mice after DMM surgery

**Fig 5:**
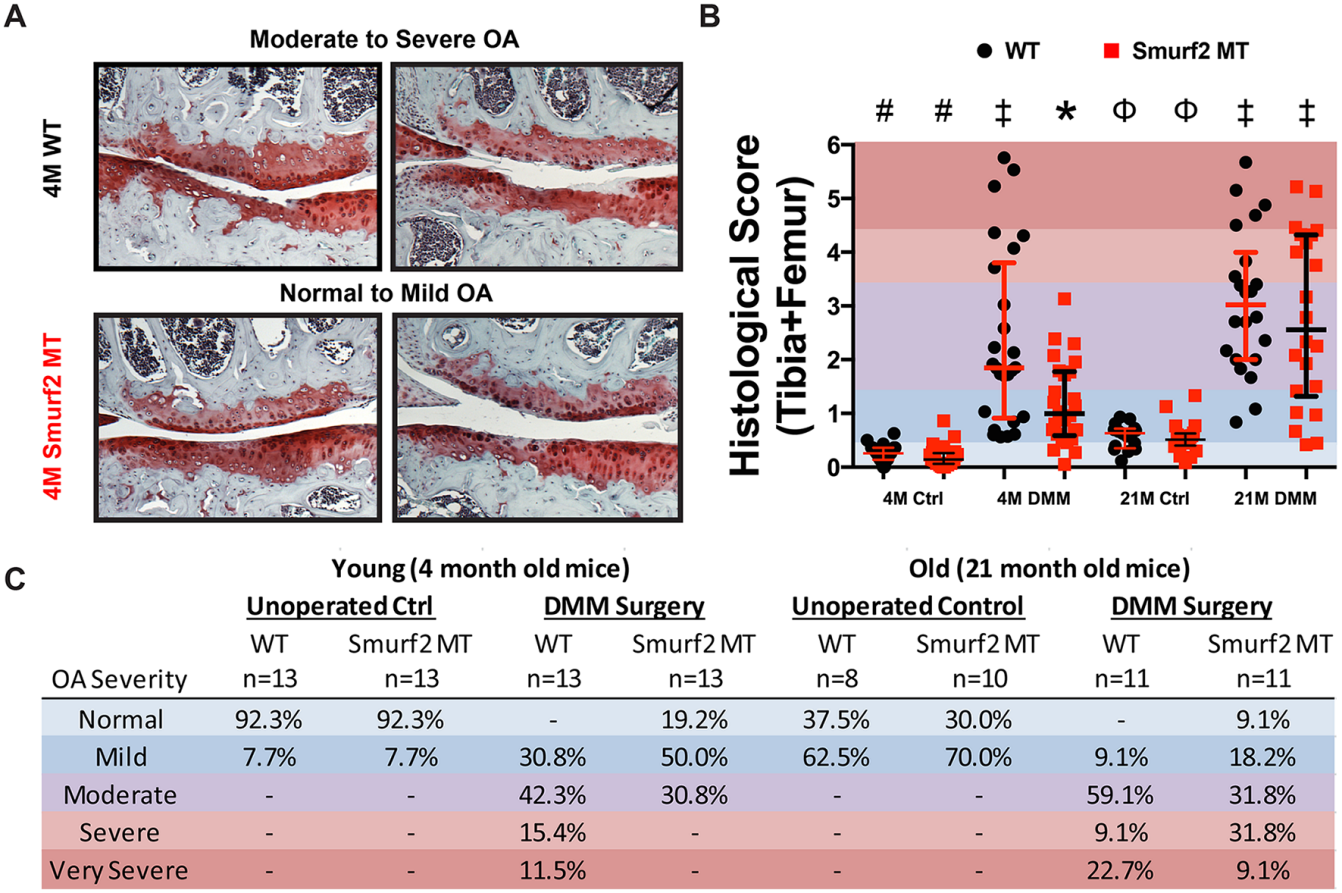
Differential severity of knee joint articular cartilage erosions in young (4 month) and old (21 ± 1.3 month) male WT and Smurf2-deficient MT mice after DMM surgery. (A) Representative images of safranin O-stained articular cartilage sections from the medial compartment of WT and MT knees 2 months post-DMM surgery. (B) Semi-quantitative histology scores of the femoral and tibial articular cartilage of DMM knees vs un-operated controls (n = 13 for 4 month; n = 11 for 21 ± 1.3 month). The average femoral and tibial articular cartilage scores for each joint were plotted separately on the same graph. Groups with the same symbol are not statistically significant (*p* > 0.05) based on mean ranks. (C) Distribution of DMM knee scores for WT and MT based on OA severity.

DMM is a well-established surgical-induced model of OA in mice that mimics the slow progression of human OA following traumatic knee injury [24]. To determine whether Smurf2 deficiency affects the pathological development and progression of OA or alter the repair mechanisms in response to knee injury, we performed DMM surgery on 2 month old male WT and MT mice and evaluated their knee articular cartilage by histological scoring after 2 months. Histological sections from the medial side of the DMM-operated knees revealed cartilage erosions extending below the superficial layer and past the tidemark in the WT mice while mainly loss of proteoglycan staining and superficial fibrillations were observed in the MT DMM knees (Fig 5A). This difference resulted in a post-DMM median histological score of 1.85 (0.91–3.80) for WT and 1.00 (0.59–1.78) for MT, both of which were significantly higher than the respective un-operated contralateral controls (*p* < 0.0001) and significantly different from each other (*p* < 0.01) (Fig 5B). We also observed a profound difference in the distribution of the post-DMM histology scores between WT and MT, which was verified using the Kolmogorov-Smirnov test (*p* = 0.043). The difference in the distribution of cartilage scores between WT and MT knees in response to DMM is best reflected by categorizing the OA severity of each scored specimen (Fig 5C). Specifically, while all WT mice developed knee OA 2 months after DMM, with 69.2% of the articular cartilage exhibiting moderate (42.3%) to severe/very severe (26.9%) OA phenotypes, 69.2% of the articular cartilage from MT mice knees developed either mild OA (50.0%) or remained normal (19.2%). Only 30.8% of the MT mice exhibited moderate OA cartilage phenotype at 2 months after DMM and none exhibited severe or very severe OA symptoms.

Since sclerosis of the subchondral bone has been detected in mice after DMM surgery, we evaluated whether Smurf2-deficiency affected the degree of sclerosis. Representative images of WT and MT DMM knees revealed increased bone mineral density localized in the medial femoral condyle and the tibial plateau at 2 months after DMM (S3A Fig). Quantitative analysis of the medial subchondral compartment of the tibia (S3B Fig) showed an increasing trend in trabecular thickness in both WT and MT. The concurrent loss of trabecular space is more apparent in the MT mice. The changes in BMD detected before and after DMM surgery in either genotype was not statistically significant, likely due to the large standard deviation among the WT subchondral bone in response to DMM.

### Aging increases severity of OA in both WT and MT knees but the subset of mice exhibiting normal or mild OA is still greater in MT

Since age is a risk factor for OA development, we performed DMM surgery on older (19 ± 1.3 month) Smurf2-deficient mice to examine whether the attenuated OA cartilage phenotype observed at 4 months may be sustained with age. Histological scores of the older knees 2 months post-DMM revealed that aging increased the overall severity of OA symptoms for both WT (p < 0.05) and MT mice (p < 0.001) (Fig 5D and 5E). The median post-DMM histological scores for the older mice were 3.02 (2.00–4.00) for WT and 2.56 (1.32–4.32) for MT, but the reduction in OA severity in MT was not statistically significant (*p* = 0.460) based on mean ranks comparison. Unlike the 4 month old mice, the distribution of histological scores were also similar between the old WT and MT mice as verified by the Kolmogorov-Smirnov test (*p* = 0.621). It is interesting to note, however, the subset of mice exhibiting normal or mild OA articular cartilage symptoms post-DMM at such an advanced age was still greater in the MT mice (27.3%) than in the WT mice (9.1%).

### Smurf2-deficient chondrocytes display elevated chondrogenic gene expression in 2D culture compared to WT

**Fig 6:**
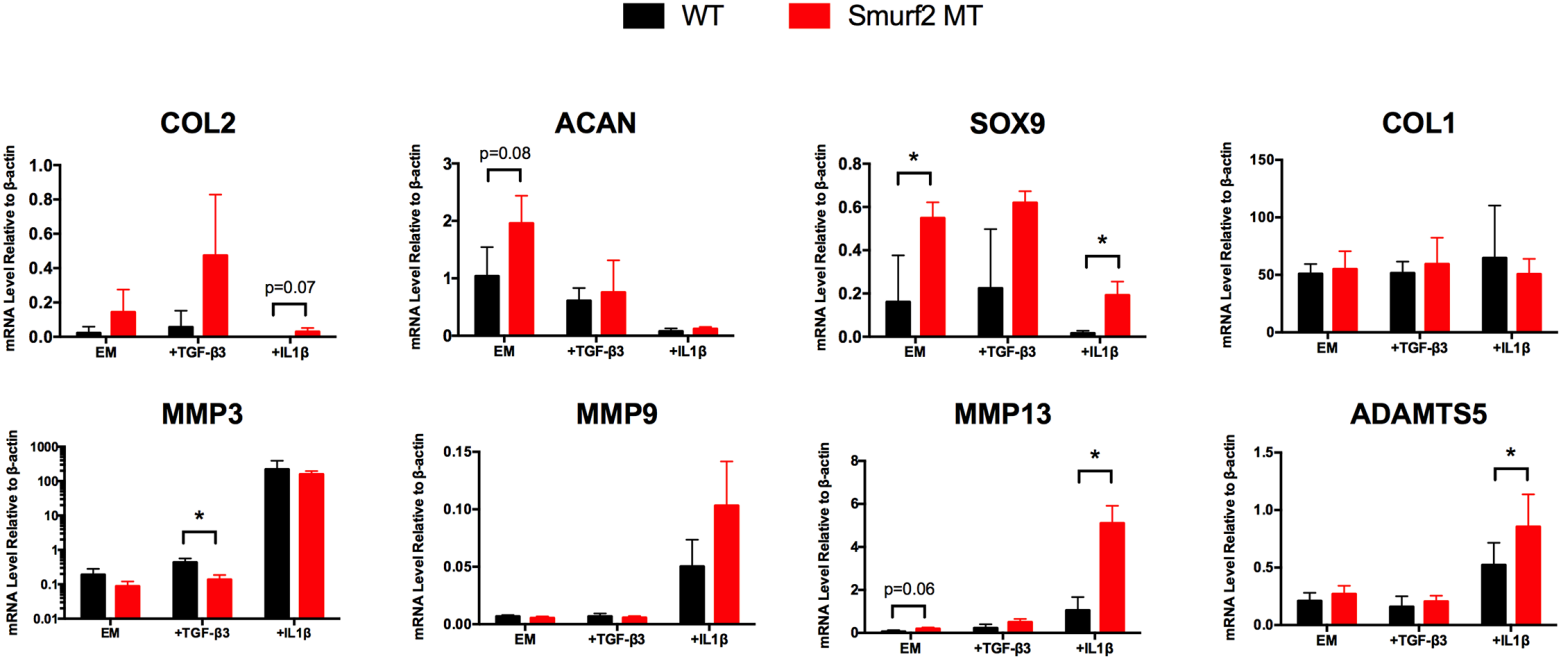
Quantitative gene expression analyses of key anabolic and catabolic markers in WT and MT iMACs. Cells were cultured as a monolayer on 2D tissue culture polystyrene for 4 days and treated with either 10 ng/mL of TGF-β3 or 5 ng/mL of IL-1β for 24 hours. Data are reported as the mean ± standard deviation of three separate chondrocyte isolations for each genotype. EM = Expansion Media, *p < 0.05 (*p* values approaching significance are noted on the graph).

To examine how Smurf2 deficiency might affect chondrocyte function in a chondrogenic or pathological environment in 2D culture, we isolated iMACs from WT and MT mice and compared the gene expression of key anabolic and catabolic genes in the presence of chondrogenic factor, TGF-β3 or proinflammatory cytokine, IL-1β. MT chondrocytes showed trend of higher levels of chondrogenic gene expression (Sox9, Col2, and Acan) than WT with and without addition of TGF-β3 (Fig 6). In the presence of pro-inflammatory cytokine IL-1β at 5 ng/mL, an overall decrease in the expression of chondrogenic genes Sox9, Col2, and Acan and an increase in the expression of catabolic genes MMP-3, MMP-9, MMP-13 and ADAMTS5 were observed in both WT and MT. It is worth noting that the trend of higher levels of chondrogenic genes in MT than WT persisted even under the inflammatory conditions, with the higher Sox9 expression in MT being statistically significant. Although there was no consistent trend in the expression of catabolic genes between WT and MT iMACs, there was a statistically significant difference in MMP13 and ADAMTS5 expression between WT and MT upon IL-1β treatment. The level of Col1, a marker for chondrocyte dedifferentiation, was similar across all conditions between genotypes. We were unable to detect Col10 expression in iMACs from either genotype after 40 cycles.

### TGF-β3 and IL-1β modulates Smurf1 and Smurf2 expression in iMACs *in vitro*

**Fig 7:**
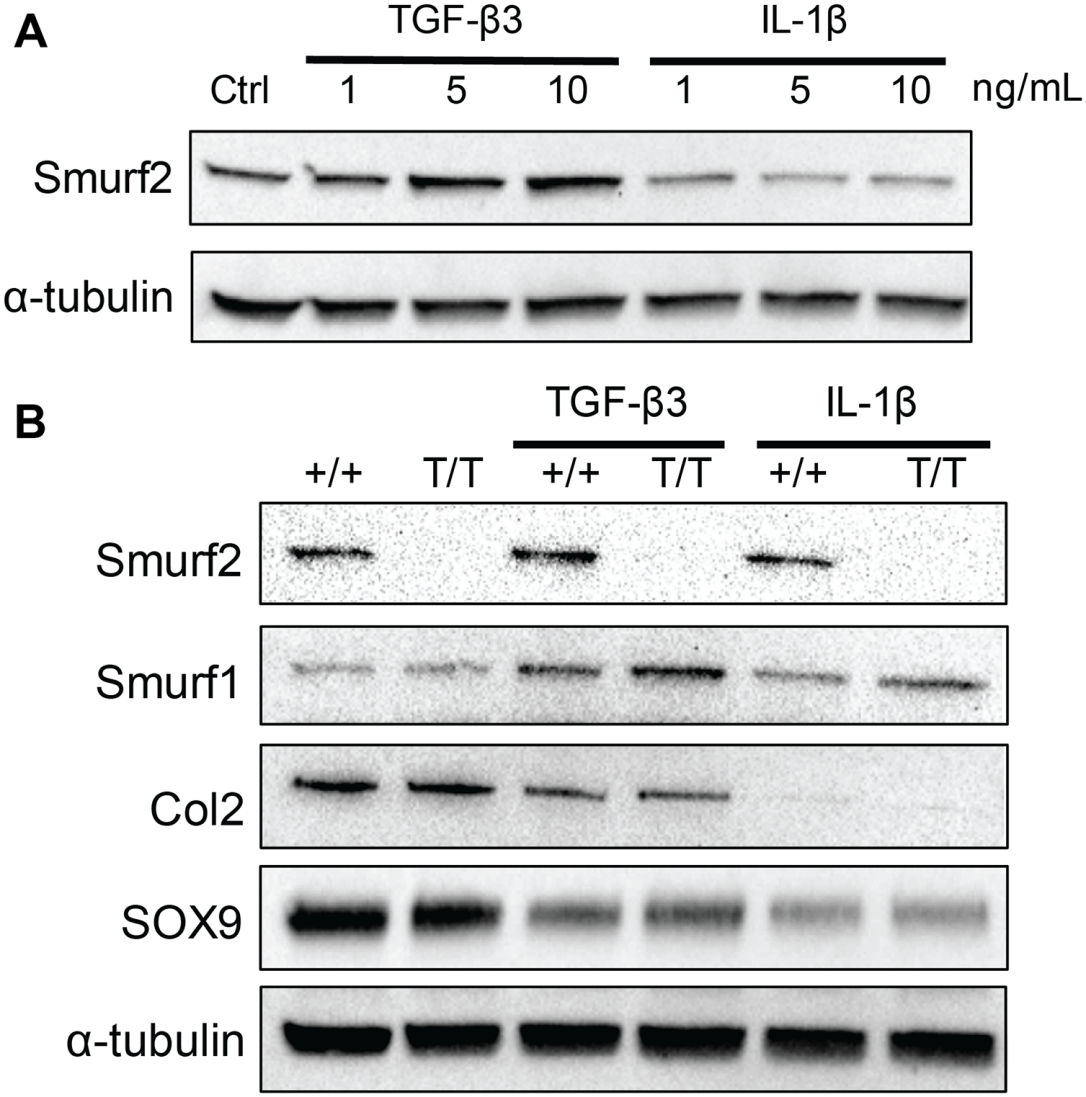
Smurfs protein expression changes upon 24-h treatment of TGF-β3 and IL-1β. A) WT chondrocytes show dose-dependent increase of Smurf2 protein with 24-h TGF-β3 treatment and dose-dependent decrease with 24-h IL-1β treatment. B) No compensatory increase of Smurf1 protein levels was detected between WT (+/+) and Smurf2-deficient MT chondrocytes (T/T) with and without treatment of TGF-β3 (10 ng/mL) or IL-1β (0.1 ng/mL). Type 2 collagen levels were also similar between WT and MT chondrocytes across all conditions.

To determine whether Smurf2 and Smurf1 protein levels are perturbed in chondrogenic or pathological conditions, we detected their expressions in WT iMACs after TGF-β3 or IL-1β treatment by western blot. Treatment of WT iMACs with TGF-β3 caused a dose-dependent increase in Smurf2 while treatment with IL-1β resulted in a dose-dependent decrease (Fig 7A). Even though the treatment of iMACs with TGF-β3 widened the level of Smurf2 between WT and MT, the difference in protein expression of Col2 and Sox9 between WT and MT were not as apparent (Fig 7B). Smurf1, which shares high homology with Smurf2, appeared slightly elevated in MT iMACs compared to WT after TGF-β3 treatment, but quantification of the intensities of these bands did not reveal statistical significance (S4 Fig).

## Discussion

In this study, we characterized bone and joint phenotypes of Smurf2-deficient mice as a function of age on the basis that Smurf1 and Smurf2 have been implicated in regulating bone and cartilage function, respectively. Using microCT and histological analyses, we compared age-dependent changes in femoral cortical bone, vertebral trabecular bone (gender-specific), tibial subchondral bone and knee articular cartilage between WT and Smurf2-deficient MT mice. We found that the reduction of Smurf2 expression did not impact the normal development and maintenance of bone and cartilage throughout adulthood and the observed bone and cartilage changes as a function of age are consistent with previous reports on aged WT C57BL/6 mice [26, 28–30].

Since the overexpression of Smurf2 in chondrocytes was reported to trigger spontaneous OA [18], we subjected skeletally mature 2 month old Smurf2-deficient mice to DMM surgery and examined whether their development of OA is mitigated compared to WT. We observed that at 2 months post-DMM, MT mice were less susceptible to pathological development of OA compared to WT. Based on our OA severity categorization criterion, the majority of articular cartilage from WT DMM knees developed moderate to severe OA (69.2%; characterized by significant cartilage fibrillations and erosions extended beyond the tidemark and into subchondral bone) while the majority of articular cartilage from MT DMM knees were normal or developed only mild OA (69.2%). More remarkably, not only did none of the articular cartilage from MT DMM knees develop severe OA, but a subset of them remained normal (19.2%). In contrast, none of the articular cartilage from WT knees were normal 2 months after DMM and a subset of them developed severe to very severe OA by 2 months (26.9%). The presence of subchondral bone sclerosis and osteophyte formation has long been associated with human OA knees. However, it remains controversial whether subchondral bone changes are an etiology of OA or whether they reflect a bone remodeling process that reacts to the deteriorating overlaying cartilage [31]. From our study, we did not detect any significant changes in subchondral bone sclerosis (S2 Fig) or osteophyte formation between genotypes by 2 months post-DMM, suggesting that the subchondral bone remodeling process induced by DMM is not entirely dependent on Smurf2 expression or the integrity of the overlaying cartilage.

Given that the impact of Smurf2 on cartilage progressed with age and that Smurf2 deficiency have been implicated to inhibit cellular senescence [19], we questioned whether the protective effect of Smuf2 deficiency against OA development after DMM surgery may sustain with age. Histological scores of the joint articular cartilage showed that aging caused both WT and MT mice to develop more severe OA after DMM surgery, with an increase of the median score for the 21 month old WT (3.02 vs. 1.85) and MT (2.56 vs. 1.00) when compared to 4 month old counterparts. Nevertheless, we found that the subset of joint cartilage that remained normal or exhibited only mild OA phenotype post-DMM is still greater among old MT than WT, suggesting that some degree of protective effect may still persist at this age. Because the majority of the specimens do develop moderate to severe OA, a larger sample size is necessary to demonstrate the significance of this subset between old WT and MT. Altered biomechanics due to weight changes and impaired bone and cartilage repair and homeostasis due to aging can all potentially mask the protective effect of Smurf2 deficiency on OA development. Overall, the effectiveness of Smurf2 inhibition to delay, alleviate or prevent OA symptoms in the multi-tissue compartments of the joint, particularly at advanced age, requires further investigation.

Taking advantage of this Smurf2-deficiency mouse model, we isolated immature articular chondrocytes from newborn mice and asked how Smurf2 deficiency affects the expression of common chondrogenic and catabolic genes upon culture treatment with TGF-β3 or IL-1β. Pro-inflammatory cytokine IL-1β is known to shift the balance of chondrocytes from matrix synthesis towards degradation and has been used to emulate an inflammatory OA environment *in vitro* [32, 33]. We found that MT chondrocytes trended toward a higher transcription level of chondrogenic genes including Sox9, Col2, and Acan compared to WT with and without TGF-β3 treatment. As expected, IL-1β treatment of the chondrocytes globally suppressed chondrogenic genes and upregulated the catabolic genes that we examined. The general trend of higher chondrogenic gene expression in the MT than WT was maintained even under proinflammatory conditions, with the difference in Sox9 being statistically significant and that of Col2 approaching significance. The differences observed between WT and MT iMACs at the mRNA level, however, did not translate directly to the protein level. We found no difference in Col2 protein expression and Sox9 protein expression was only slightly higher in the MT iMACs treated with TGF-β3 compared to WT. Interestingly, both Col2 and Sox9 expression seems to decrease after 24 hour treatment with TGF-β3 compared to untreated cells. We confirmed that this increase in chondrogenic gene expression was not due to differences in the degree of dedifferentiation between WT and MT *in vitro* by demonstrating that Col1 expression was similar across all experimental conditions. On the contrary, there was no consistent trend in the expression of catabolic genes examined (MMP-3, -9, -13 and ADAMTS5) between WT and MT. Compared to WT iMACs, MT iMACs had statistically significant lower expression of MMP3 upon TGF-β3 treatment, but higher expression of MMP-13 and ADAMTS5 after IL-1β treatment. In the absence of TGF-β3 or IL-1β, no significant differences in any catabolic genes were detected. Incidentally, we found that both TGF-β3 and IL-1β modulated Smurf2 levels in WT iMACs in a dose-dependent manner. TGF-β3 also increased Smurf1 levels and the increase was higher in MT chondrocytes than in WT, suggesting a potential but weak compensatory mechanism by Smurf1 in Smurf2-deficient iMACs. Whether TGF-β3-induced increase of Smurf1 and Smurf2 affects the phosphorylation of intracellular R-Smads or the stability of TGF-β receptors or other downstream effector proteins remain to be determined.

Despite previous reports demonstrating the effect of Smurf to modulate the TGF-β/BMP signaling pathway [34, 35] and the importance of this pathway in bone and cartilage development [36–38], the lack of an obvious skeletal phenotype in MT mice during normal development and aging as elucidated by this study has a few implications. It suggests that a high level of Smurf2 expression may not be required for bone and cartilage development and that the low levels of Smurf2 present in the MT mice may be sufficient to maintain normal physiological activities. In addition, Smurf1 may have redundant functions that can compensate for the reduced Smurf2 levels during normal development and aging, and account for the moderate and weak protection against OA development observed in young and old Smurf2-deficient mice, respectively.

There are a few limitations of our study. First, it is unclear whether reduced Smurf2 expression functions to delay the onset of OA and/or hinders its progression. We have not identified the exact stress signals produced by DMM that can trigger Smurf2 activity or stimulate its expression beyond physiological levels, which could help establish a molecular basis of how Smurf2 drives OA pathogenesis. Second, besides articular cartilage and subchondral bone, other joint tissue compartments involved in OA pathogenesis such as synovium, muscles and bone marrow were not examined in this study. Third, the use of immature articular chondrocytes, driven by its ease of isolation, to compare the chondrogenic potential of WT and MT cells may not be ideal. Not only is the population of iMACs heterogenous with varying degrees of differentiation, but their response to exogenous growth factors and cytokines may be different from the chondrocytes residing in matured articular cartilage. Tissue-specific Smurf2-deficient cells (e.g. obtained via conditional knockouts) may be more accurate at correlating the potential cellular contributors within the multi-tissue joint compartment to the protective OA phenotype observed in MT mice after DMM. Lastly, to unequivocally establish the redundant function of Smurf1 and Smurf2 in chondrocytes, validation by simultaneous knock down experiments would be desired.

In summary, we show that while loss of Smurf2 in mice has no physiological impact on bone and cartilage development and aging, its ability to attenuate OA symptoms after DMM surgery in 4 month old mice indicate a potential role in regulating the pathological development of OA. Whether Smurf2 inhibition alone is sufficient to be a therapeutic target for OA remains unclear since the protective effect of Smurf2 in older mice is markedly reduced compared to the younger age group. The enhanced chondrogenic gene expressions in MT iMACs compared to WT suggest a potential for enhanced cartilage matrix deposition, but the optimal condition to facilitate its translation into meaningful enhancements in cartilage regeneration remain to be identified.

## Supporting Information

S1 Fig ROI Definitions of microCT Analysis. (A) ROI within L4/L5 vertebrae indicated by the green contour; (B) ROI within the lateral and medial compartments of tibial subchondral bone indicated by green and red contours, respectively. Contour of medial compartment excluded osteophyte projection and the overlying calcified cartilage.
(TIF)

S2 Fig Representative safranin O-stained knee joint histological sections of young and old WT versus Smurf2-deficient MT mice. (TIF)

S3 Fig Knee joint subchondral bone analyses in 4 months old male WT and Smurf2-deficient MT mice upon surgical induction of OA by DMM. A) Representative microCT bone mineral density (BMD) color mapping of medial compartment of 4 month old male mice knee 2 months post DMM. White arrows = areas with increased bone mineral content; L = lateral; M = medial; red indicates higher BMD while green indicates lower BMD. B) Quantification of subchondral bone change in medial compartment of tibial plateau after DMM reflecting trends of increased BV/V, trabecular thickness and decreased trabecular spaces. WT: n = 9; MT: n = 11.
(TIF)

S4 Fig Western blot quantification of Smurf2 and Smurf1 proteins in WT and MT iMACs. Protein bands for Smurf2 and Smurf1 bands were quantified and normalized to WT iMAC in expansion media (EM). Quantification was based on the average of three separate experiments with different WT and MT pairs. n.d. = not detected.
(TIFF)

## References

[1] Chen G, Deng C, Li Y-P. TGF-β and BMP Signaling in Osteoblast Differentiation and Bone Formation. Int J Biol Sci 2012;8(2):272–288. 10.7150/ijbs.2929 22298955 PMC3269610

[2] Redini F, Galera P, Mauviel A, Loyau G, Pujol JP. Transforming growth factor beta stimulates collagen and glycosaminoglycan biosynthesis in cultured rabbit articular chondrocytes. FEBS Lett 1988;234(1):172–6. 3164687 10.1016/0014-5793(88)81327-9

[3] Yang X, Chen L, Xu X, Li C, Huang C, Deng CX. TGF-beta/Smad3 signals repress chondrocyte hypertrophic differentiation and are required for maintaining articular cartilage. J Cell Biol 2001;153(1):35–46. 11285272 10.1083/jcb.153.1.35 PMC2185521

[4] Rahman MS, Akhtar N, Jamil HM, Banik RS, Asaduzzaman SM. TGF-β/BMP signaling and other molecular events: regulation of osteoblastogenesis and bone formation. Bone Res 2015;3:15005 10.1038/boneres.2015.5 26273537 PMC4472151

[5] Bauge C, Girard N, Lhuissier E, Bazille C, Boumediene K. Regulation and Role of TGF-β Signaling Pathway in Aging and Osteoarthritis Joints. Aging Disease 2014;5(6):394–405. 10.14336/AD.2014.0500394 25489490 PMC4249809

[6] Verdier MP, Seité S, Guntzer K, Pujol JP, Boumédiène K. Immunohistochemical analysis of transforming growth factor beta isoforms and their receptors in human cartilage from normal and osteoarthritic femoral heads. Rheumatol Int 2005;25(2):118–24. 14618374 10.1007/s00296-003-0409-x

[7] Blaney Davidson EN, Vitters EL, van der Kraan PM, van den Berg WB. Expression of transforming growth factor-beta (TGFbeta) and the TGFbeta signalling molecule SMAD-2P in spontaneous and instability-induced osteoarthritis: role in cartilage degradation, chondrogenesis and osteophyte formation. Ann Rheum Dis 2006;65(11):1414–21. 16439443 10.1136/ard.2005.045971 PMC1798346

[8] Serra R, Johnson M, Filvaroff EH, LaBorde J, Sheehan DM, Derynck R, et al Expression of a truncated, kinase-defective TGF-beta type II receptor in mouse skeletal tissue promotes terminal chondrocyte differentiation and osteoarthritis. J Cell Biol 1997;139(2):541–52. 9334355 10.1083/jcb.139.2.541 PMC2139797

[9] Blaney Davidson EN, Scharstuhl A, Vitters EL, van der Kraan PM, van den Berg WB. Reduced transforming growth factor-beta signaling in cartilage of old mice: role in impaired repair capacity. Arthritis Res Ther 2005;7(6):R1338–47. 16277687 10.1186/ar1833 PMC1297583

[10] Li TF, Gao L, Sheu TJ, Sampson ER, Flick LM, Kontinnen YT, et al Aberrant hypertrophy in Smad3-deficient chondrocytes is rescued by restoring transforming growth factor beta-activated kinase 1/activating transcription factor 2 signaling: a potential clinical implication for osteoarthritis. Arthritis Rheum 2010;62(8):2359–69. 10.1002/art.27537 20506210 PMC2921996

[11] Hershko A and Ciechanover A. The ubiquitin system. Ann Rev Biochem 1998;67:425–79. 9759494 10.1146/annurev.biochem.67.1.425

[12] Tang LY, Yamashita M, Coussens NP, Tang Y, Wang X, Li C, et al Ablation of Smurf2 reveals an inhibition in TGF-β signalling through multiple mono-ubiquitination of Smad3. EMBO J. 2011;30(23):4777–89. 10.1038/emboj.2011.393 22045334 PMC3243605

[13] Jin L, Pahuja KB, Wickliffe KE, Gorur A, Baumgartel C, Schekman R, et al Ubiquitin-dependent regulation of COPII coat size and function. Nature 2012 2 22;482(7386):495–500. 10.1038/nature10822 22358839 PMC3292188

[14] Inoue Y and Imamura T. Regulation of TGF-β family signaling by E3 ubiquitin ligases. Cancer science 2008; 99(11): 2107–12. 10.1111/j.1349-7006.2008.00925.x 18808420 PMC11158544

[15] Bizet AA, Tran-Khanh N, Saksena A, Liu K, Buschmann MD, Philip A. CD109-mediated degradation of TGF-β receptors and inhibition of TGF-β responses involve regulation of SMAD7 and Smurf2 localization and function. J Cell Biochem 2012 1;113(1):238–46. 10.1002/jcb.23349 21898545

[16] Xing L, Zhang M, Chen D. Smurf control in bone cells. J Cell Biochem 2010;110(3):554–563. 10.1002/jcb.22586 20512916 PMC2886145

[17] Yamashita M, Ying SX, Zhang GM, Li C, Cheng SY, Deng CX, et al Ubiquitin ligase Smurf1 controls osteoblast activity and bone homeostasis by targeting MEKK2 for degradation. Cell 2005;121(1):101–13. 15820682 10.1016/j.cell.2005.01.035 PMC3314294

[18] Wu Q, Kim KO, Sampson ER, Chen D, Awad H, O'Brien T, et al Induction of an osteoarthritis-like phenotype and degradation of phosphorylated Smad3 by Smurf2 in transgenic mice. Arthritis Rheum 2008;58(10):3132–44. 10.1002/art.23946 18821706 PMC2636703

[19] Ramkumar C, Kong Y, Cui H, Hao S, Jones SN, Gerstein RM, et al Smurf2 regulates the senescence response and suppresses tumorigenesis in mice. Cancer Res 2012;72(11):2714–9. 10.1158/0008-5472.CAN-11-3773 22552287 PMC3616636

[20] Blank M, Tang Y, Yamashita M, Burkett SS, Cheng SY, Zhang YE. A tumor suppressor function of Smurf2 associated with controlling chromatin landscape and genome stability through RNF20. Nat Med 2012;18(2):227–34. 10.1038/nm.2596 22231558 PMC3274650

[21] Suire C, Brouard N, Hirschi K, Simmons PJ. Isolation of the stromal-vascular fraction of mouse bone marrow markedly enhances the yield of clonogenic stromal progenitors. Blood 2012;119(11):e86–95. 10.1182/blood-2011-08-372334 22262767 PMC4507041

[22] Gosset M, Berenbaum F, Thirion S, Jacques C. Primary culture and phenotyping of murine chondrocytes. Nat Protoc 2008;3(8):1253–60. 10.1038/nprot.2008.95 18714293

[23] Glasson SS, Chambers MG, Van Den Berg WB, Little CB. The OARSI histopathology initiative—recommendations for histological assessments of osteoarthritis in the mouse. Osteoarthritis Cartilage 2010;18 Suppl 3:S17–23. 10.1016/j.joca.2010.05.025 20864019

[24] Glasson SS, Blanchet TJ, Morris EA. The surgical destabilization of the medial meniscus (DMM) model of osteoarthritis in the 129/SvEv mouse. Osteoarthritis Cartilage 2007;15(9):1061–9. 17470400 10.1016/j.joca.2007.03.006

[25] Seeman E. Age- and menopause-related bone loss compromise cortical and trabecular microstructure. J Gerontol A Biol Sci Med Sci 2013;68(10):1218–25. 10.1093/gerona/glt071 23833200

[26] McNulty MA, Loeser RF, Davey C, Callahan MF, Ferguson CM, Carlson CS. Histopathology of Naturally Occurring and Surgically Induced Osteoarthritis in Mice. Osteoarthritis Cartilage 2012;20(8):949–956. 10.1016/j.joca.2012.05.001 22595226 PMC3402508

[27] Stok KS, Pelled G, Zilberman Y, Kallai I, Goldhahn J, Gazit D, et al Revealing the interplay of bone and cartilage in osteoarthritis through multimodal imaging of murine joints. Bone 2009;45(3):414–22. 10.1016/j.bone.2009.05.017 19481620

[28] Halloran BP, Ferguson VL, Simske SJ, Burghardt A, Venton LL, Majumdar S. Changes in bone structure and mass with advancing age in the male C57BL/6J mouse. J Bone Miner Res 2002;17(6):1044–50. 12054159 10.1359/jbmr.2002.17.6.1044

[29] Glatt V, Canalis E, Stadmeyer L, Bouxsein ML. Age-Related Changes in Trabecular Architecture Differ in Female and Male C57BL/6J Mice. J Bone Miner Res 2007;22(8).1197–1207. 17488199 10.1359/jbmr.070507

[30] Yamamoto K, Shishido T, Masaoka T, Imakiire A. Morphological studies on the ageing and osteoarthritis of the articular cartilage in C57 black mice. J Orthop Surg 2005;13(1):8–18. 10.1177/230949900501300103 15872395

[31] Burr DB, Gallant MA. Bone remodelling in osteoarthritis. Nat Rev Rheumatol 2012;8:665–673. 10.1038/nrrheum.2012.130 22868925

[32] Tetlow LC, Adlam DJ, Wooley DE. Matrix metalloproteinase and proinflammatory cytokine production by chondrocytes of human osteoarthritic cartilage. Arthritis Rheum 2001;44(3):585–94. 11263773 10.1002/1529-0131(200103)44:3<585::AID-ANR107>3.0.CO;2-C

[33] Rowan AD and Young DA. Collagenase gene regulation by pro-inflammatory cytokines in cartilage. Frontiers in Bioscience. 2007;12:536–50. 17127315 10.2741/2080

[34] Lin X, Liang M, Feng XH. Smurf2 is a ubiquitin E3 ligase mediating proteasome-dependent degradation of Smad2 in transforming growth factor-beta signaling. J Biol Chem 2000;275(47):36818–22. 11016919 10.1074/jbc.C000580200

[35] Kavsak P, Rasmussen R, Causing C, Bonni S, Zhu H, Thomsen GH, et al Smad7 Binds to Smurf2 to Form an E3 Ubiquitin Ligase that Targets the TGF-β Receptor for Degradation. Mol Cell 2000;6:1365–1375. 11163210 10.1016/s1097-2765(00)00134-9

[36] Blaney Davidson EN, van der Kraan PM, van den Berg WB. TGF-beta and osteoarthritis. Osteoarthritis Cartilage 2007;15(6):597–604. 17391995 10.1016/j.joca.2007.02.005

[37] Wu Q, Wang M, Zuscik MJ, Chen D, O'Keefe RJ, Rosier RN. Regulation of embryonic endochondral ossification by Smurf2. J Orthop Res 2008;26(5):704–12. 10.1002/jor.20563 18176945 PMC2636972

[38] Keller B, Yang T, Chen Y, Munivez E, Bertin T, et al Interaction of TGFβ and BMP signaling pathways during chondrogenesis. PLoS One 2011;6(1)e16421:1–9. 10.1371/journal.pone.0016421 21297990 PMC3030581

