# Supplementary figures and images for: Skeletal Characterization of Smurf2-Deficient Mice and In Vitro Analysis of Smurf2-Deficient Chondrocytes
Source: PLoS One. 2016 Jan 27;11(1):e0148088. doi: 10.1371/journal.pone.0148088 (PMC4729489; doi:10.1371/journal.pone.0148088)

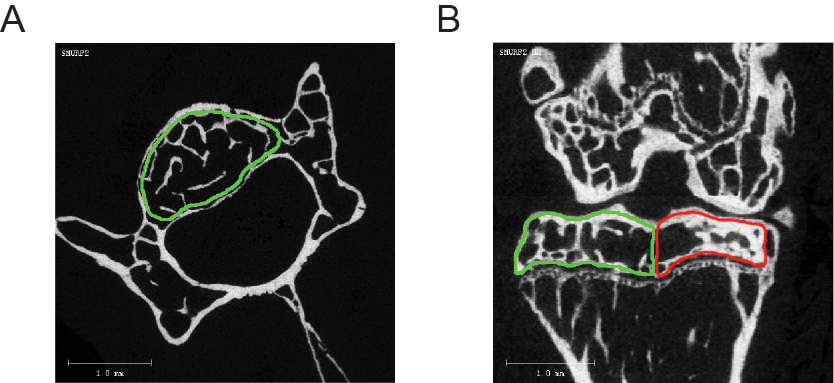

Supplement: S1 Fig — (A) ROI within L4/L5 vertebrae indicated by the green contour; (B) ROI within the lateral and medial compartments of tibial subchondral bone indicated by green and red contours, respectively. Contour of medial compartment excluded osteophyte projection and the overlying calcified cartilage. (TIF) [file pone.0148088.s001.tif]

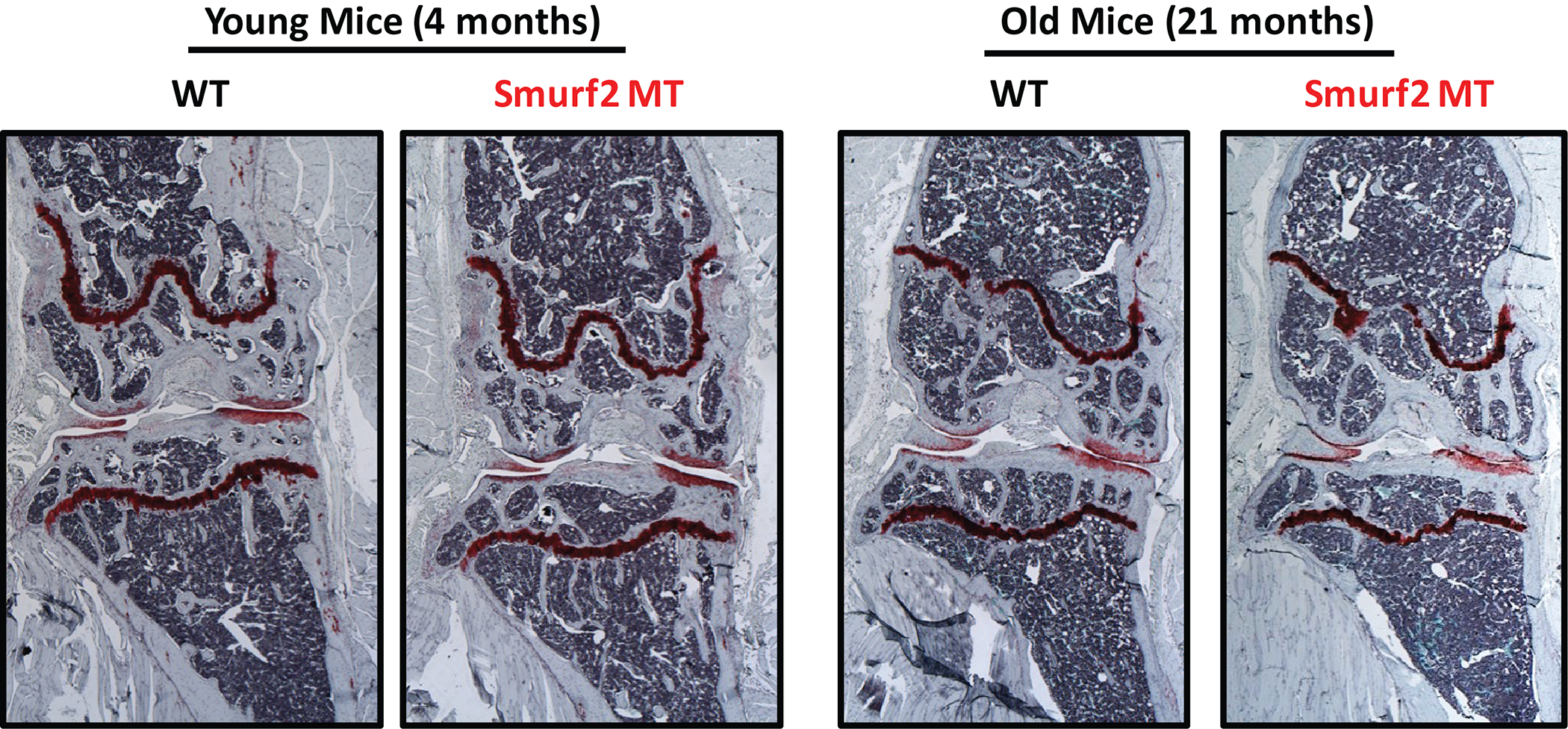

Supplement: S2 Fig — (TIF) [file pone.0148088.s002.tif]

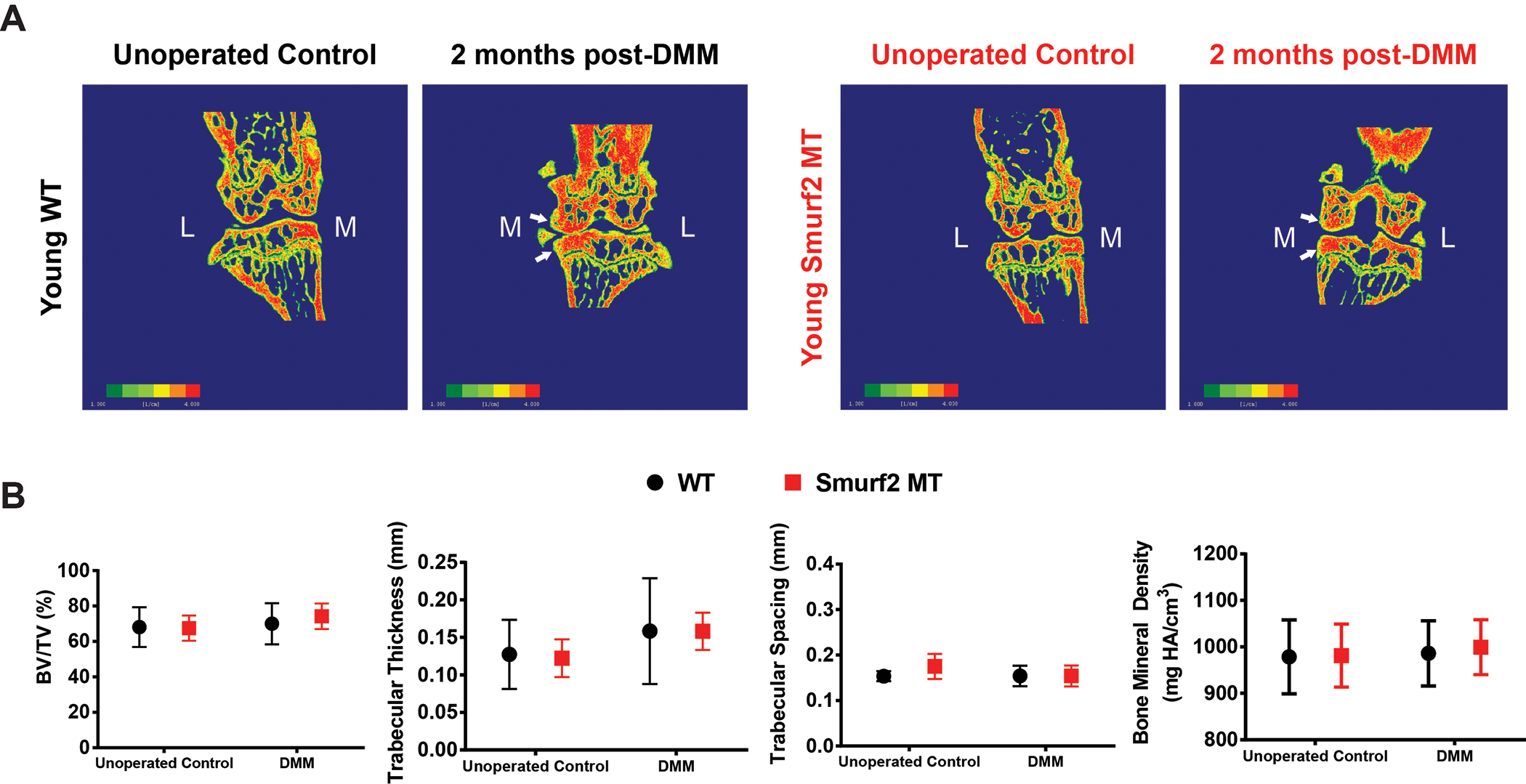

Supplement: S3 Fig — A) Representative microCT bone mineral density (BMD) color mapping of medial compartment of 4 month old male mice knee 2 months post DMM. White arrows = areas with increased bone mineral content; L = lateral; M = medial; red indicates higher BMD while green indicates lower BMD. B) Quantification of subchondral bone change in medial compartment of tibial plateau after DMM reflecting trends of increased BV/V, trabecular thickness and decreased trabecular spaces. WT: n = 9; MT: n = 11. (TIF) [file pone.0148088.s003.tif]

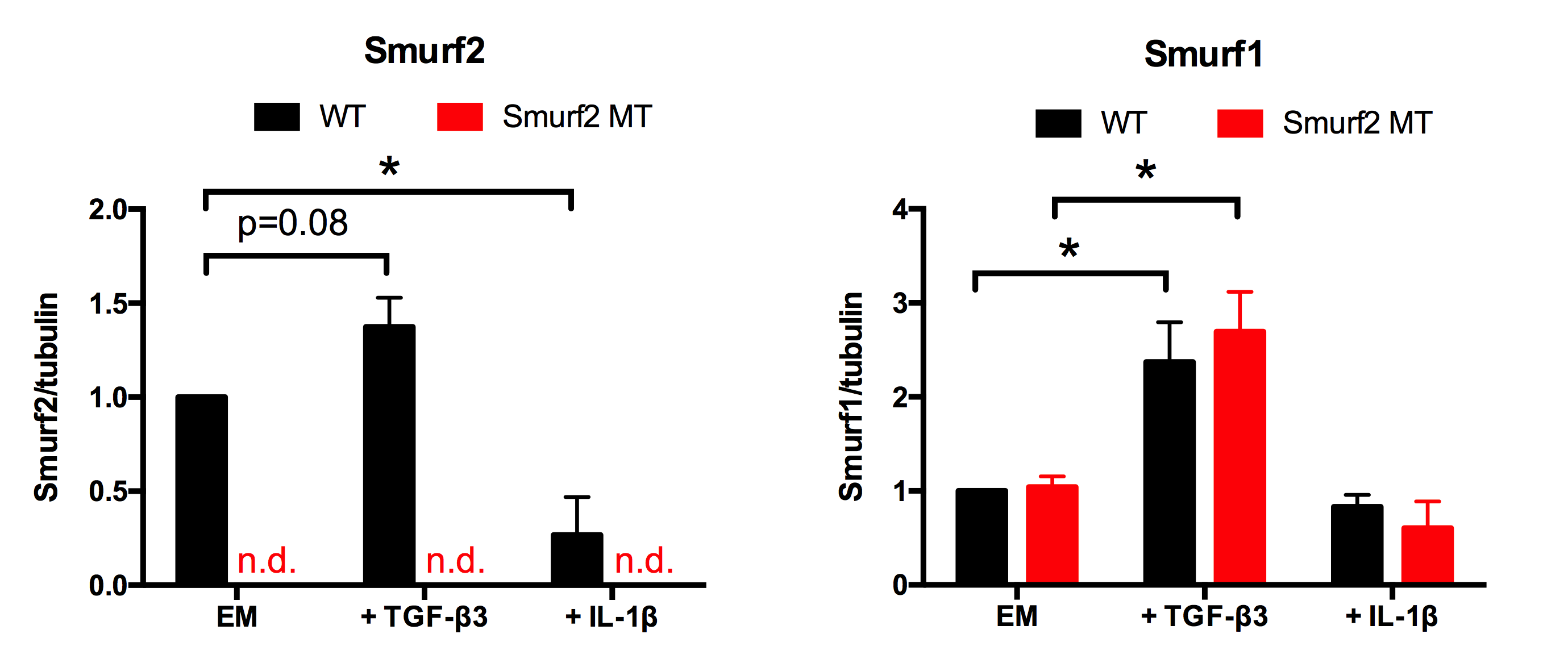

Supplement: S4 Fig — Protein bands for Smurf2 and Smurf1 bands were quantified and normalized to WT iMAC in expansion media (EM). Quantification was based on the average of three separate experiments with different WT and MT pairs. n.d. = not detected. (TIFF) [file pone.0148088.s004.tiff]
